# Effects of an individualised exercise programme plus Behavioural Change Enhancement (BCE) strategies for managing fatigue in frail older adults: a cluster randomised controlled trial

**DOI:** 10.1186/s12877-023-04080-0

**Published:** 2023-06-16

**Authors:** Justina Y. W. Liu, Yue-Heng Yin, Patrick P. K. Kor, Rick Y. C. Kwan, Paul H. Lee, Wai Tong Chien, Parco M. Siu, Keith D. Hill

**Affiliations:** 1 Centre for Gerontological Nursing, School of Nursing, The Hong Kong Polytechnic University, Hong Kong SAR, China; 2 Research Institute of Smart Ageing, The Hong Kong Polytechnic University, Hong Kong SAR, China; 3 Southampton Clinical Trials Unit, University of Southampton, Southampton, UK; 4 Nethersole School of Nursing, The Chinese University of Hong Kong, Shatin, NT, Hong Kong SAR China; 5 Division of Kinesiology, School of Public Health, The University of Hong Kong, Hong Kong SAR, China; 6 Rehabilitation Ageing and Independent Living (RAIL) Research Centre, School of Primary and Allied Health Care, Monash University, Melbourne, 3800 Australia

**Keywords:** Fatigue, Frailty, Exercise, Behaviour change enhancement

## Abstract

**Background:**

To the best of our knowledge, although ageing-induced fatigue could cause adverse outcomes such as frailty, there is currently no intervention for it. This study evaluated the effects of an individualised exercise programme with/without BCE strategies on reducing fatigue in older adults.

**Methods:**

A three-armed cluster-randomised controlled trial (RCT) was conducted with 184 participants (mean age: 79.1 ± 6.4; mean frailty score: 2.8 + 0.8) from 21 community centres (ClinicalTrials.gov: NCT03394495). They were randomised into either: the COMB group (*n* = 64), receiving 16 weeks of exercise training plus the BCE programme; the EXER group (*n* = 65), receiving exercise training and health talks; or the control group (*n* = 55), receiving only health talks. Fatigue was assessed using the Multi-dimensional Fatigue Inventory (range: 20 to 100, with higher scores indicating higher fatigue levels) at baseline, and immediately, 6 months, and 12 months post-intervention.

**Results:**

The GEE analyses showed significant interaction (time x group) between the COMB and control groups immediately (*p* < 0.001), 6 months (*p* < 0.001), and 12 months (*p* < 0.001) post-intervention. Comparing the COMB and EXER groups, there was a significant interaction immediately (*p* = 0.013) and at 12 months post-intervention (*p* = 0.007). However, no significant difference was seen between the EXER group and control group at any time point.

**Conclusions:**

The COMB intervention showed better immediate and sustainable effects (i.e., 12 months after the intervention) on reducing fatigue in frail older adults than exercise training or health education alone.

**Trial registration:**

ClinicalTrials.gov (NCT03394495), registered on 09/01/2018.

**Supplementary Information:**

The online version contains supplementary material available at 10.1186/s12877-023-04080-0.

## Background

Fatigue caused by the physiological changes of ageing is a common, self-reported, debilitating symptom among older adults, affecting their physical and psychological wellbeing [1]. The occurrence of fatigue in older adults involves a complex interplay of medical, physical, and psychiatric factors, but age-related physiological changes play a major role in its manifestation. It is known as ageing-related fatigue [2]. In this paper, fatigue is used to refer to ageing-related fatigue as it is a common complaint among older adults and is often related to the ageing process [2]. The prevalence of fatigue among older adults is around 15.3% to 31.2% worldwide [3]. Fatigue is one of the main features of frailty [4]. It can lead to restricted physical activity, poorer functioning, an increased risk of morbidity, and even to mortality in the long term [2, 5–7].

Physical exercise training, a commonly used non-pharmacological intervention, has effectively reduced ageing-related fatigue [8]. For example, a recent systematic review [9] concluded that some non-pharmacological interventions such as mindfulness, muscle rel﻿axation, pet therapy, yoga, and Tai Chi appeared to be effective in alleviating fatigue immediately post-intervention in community-dwelling older people with ageing-induced fatigue. The interventions included in this review mainly relate to physical-mental training. For example, yoga and Tai Chi mainly aim to bring body and mind into harmony, increase muscle tone, and relieve nervous and muscular tension, resulting in increased energy levels [10, 11]. This mechanism may be different from other forms of physical exercise training such as resistance muscle training or aerobic exercise. The mechanism of physical exercise training on fatigue may work by reducing inflammation, decreasing neurodegeneration, and normalising hypothalamic–pituitary–adrenal function [12]. Studies investigating the effects of physical exercise on the fatigue experienced by older adults are currently limited if not lacking, particularly those on the sustainable effects of physical exercise [13].

Older adults with fatigue may avoid exercising because physical overexertion may induce muscle pain and weakness. This explains why older adults frequently cite fatigue as a cause of their non-adherence to or withdrawal from exercise programmes [13, 14]. Their inability to function at previous levels may lead to frustration and low motivation, self-esteem, and self-efficacy in their daily activities, which in turn become major barriers to their engagement in regular exercise [15]. However, avoiding exercise exacerbates fatigue-related physiological symptoms (e.g., reduced physical endurance, mobility, and cardio-respiratory functions) [14, 16]. These physiological changes, which result from deconditioning and avoidance of physical activity, can lead to a self-perpetuating vicious cycle of fatigue [14, 16].

Studies have shown that personalised behavioural strategies could provide approaches to dealing with problems of motivation, goal setting, cognitive restrictions, and social support for sedentary older adults, leading to an increase in their self-efficacy, control beliefs, and adherence to exercise programmes [17–19]. Therefore, it is recommended that behavioural change enhancement (BCE) strategies be used along with exercise training. The health action process approach (HAPA) is a well-developed social cognition model that is used to guide BCE strategies [20]. This model contains motivational and volitional phases, and it builds a bridge to overcome the ‘intention-behaviour gap’ by applying the constructs of action planning and coping planning during the change process. Previous studies have shown that interventions guided by the HAPA model effectively promoted adherence to physical activity in different populations, including older adults [21, 22].

Therefore, this study aimed to evaluate whether an individualised exercise programme with BCE strategies guided by the HAPA model could enhance self-efficacy in complying with an exercise regime, leading to a reduction in fatigue among frail older people. It was hypothesised that participants who received a combined intervention of individualised exercise and the BCE intervention (COME), and those who received individualised exercise (EXER) would demonstrate a significant reduction in fatigue immediately after the 16-week intervention (T1), and at 6 months (T2) and 12 months (T3) post-intervention when compared with the control (health talks) groups. However, the reduction in fatigue would be more significant and more sustainable in the COME group compared to the EXER and control groups at all measurement time points (T1-3).

## Methods

### Trial design

A cluster randomised controlled trial (RCT) with three parallel groups was adopted in this study to test the hypothesis. The methods employed in the trial followed the CONSORT Cluster Trials Checklist [23] and are reported in this paper.

### Settings

The clusters were district community or neighbourhood centres for older people in Hong Kong funded by and under the supervision of the Hong Kong Government. The major purpose of these centres is to provide community support services, such as health education, counselling services, meal services, and social and recreational activities to individuals aged 65 or older and their care givers [24]. An invitation letter was sent to all government-funded community centres for older people (*n* = 211) in Hong Kong. Eventually, 21 centres accepted the invitation to join the study.

### Eligibility criteria for participants

The target population of this study was community-dwelling older people who were frail and had a nonspecific cause of fatigue. This type of fatigue does not have an identifiable medical or psychological cause. It is a common complaint among people of all ages and can be caused by a variety of factors, such as poor sleep quality, stress, lack of physical activity, dehydration, poor nutrition, or medication side effects [25, 26]. Participants were recruited through the community centres from January 2018 to April 2020. The criteria for the inclusion of participants were: 1) community-dwelling individuals aged 70 years or older; 2) able to communicate in Cantonese to ensure that they could understand the instructions; 3) able to walk with or without an assistive device to complete the Timed-Up-and-Go (TUG) test [27] because adequate mobility and balance are needed to complete the exercise training; and 4) in a pre-frail or frail status with exhaustion as determined by a Fried Frailty Index (FFI) score of 1–5, with 1–2 indicating pre-frailty and > 3 indicating frailty [28].

Older adults were excluded if they had: 1) any disease in which fatigue was a dominant symptom (e.g., neurodegenerative diseases, cancer, and end-stage renal failure cachexia; 2) been hospitalised for at least five days in the preceding three months, likely leading to muscle wasting due to recent bed rest or reduced activity levels during hospitalisation; 3) been confined to bed or restricted by the permanent use of a wheelchair; 4) been regularly performing moderate and/or intense exercise (such as hiking [29] and Tai Chi) for > 3 h per week; 5) a terminal illness; 6) a clinical diagnosis of major depression with frequent adjustments of antidepressants to control depressive mood and depression-induced fatigue; and 7) major surgery (such as a hip fracture or major abdominal surgery) in the past six months.

### Intervention

**Fig. 1:**
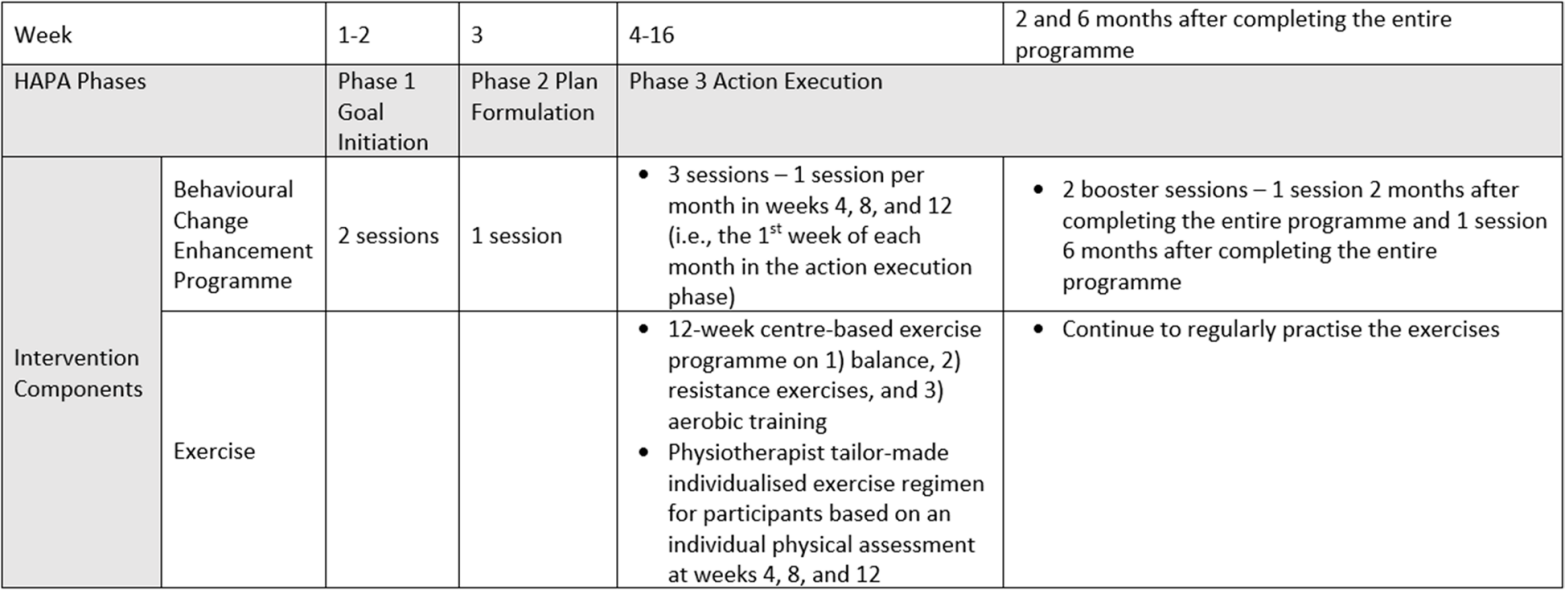
Overview of the intervention for the COMB group

The interventions started from March 2018 to January 2020. The last booster session ended in July 2020. The COMB (i.e., the experimental) group received a 16-week programme with a combination of individualised exercise training and the BCE programme. The EXER group (i.e., the active control) received another 16-week programme with a combination of exercise and health talks. To understand the change in the fatigue levels and other physio-psychosocial parameters of our target population, in this study the control group (i.e., a passive control) received health talks with no other intervention. To control the group and social interaction effects of the BCE programme for the COMB group, the number and timing of the health talks for the other two groups were similar to those in the BCE sessions. That means that one health talk per week was arranged from weeks 1–3, and then one per month in weeks 4, 8, and 12, and one 2 months and 6 months after the completion of the entire programme, respectively. The detailed intervention protocol has been published elsewhere [30]. There was no change in the implementation compared with the original protocol. Below is a brief description of the intervention for the COMB group (Fig. 1).

### Behavioural Change Enhancement (BCE) Programme in the COMB group

The Health Action Process Approach (HAPA) model was used as a framework to design the BCE programme [31]. Thus, the programme was divided into three phases based on the HAPA model:

- *Phase I Goal Initiation* was aimed at motivating the participants to develop a goal to actively manage their fatigue. ‘Increased risk awareness’, ‘enhanced self-efficacy’, and ‘improved awareness of outcome expectancies’ were embedded in the contents to motivate individuals to initiate goals to modify their behaviours [31, 32].
- *Phase II Plan Formulation* was aimed at guiding the participants in transforming their goals into detailed individualised action plans. Specifically, they were guided to set specific, measurable, achievable, realistic, and time-based (SMART) goals [33]. For instance, the COMB and EXER groups received monthly physiotherapy sessions in which a physiotherapist evaluated each participant's adherence to and performance of their customised exercise programme. The physiotherapist then tailored each participant's protocol based on their progress and needs. Although all participants were doing similar types of muscle resistance training exercise, the number of repetitions and the weight of the resistant bands differed based on the individual’s performance. Therefore, the participants adjusted their personal goals and individualised their action plan according to the physiotherapist’s recommendation. A coping plan was also formed with reference to the anticipated barriers, and alternative plans were generated to overcome those barriers. Both plans and (sub-) goals were modified based on the individual’s progress throughout the programme.
- *Phase III Action Execution and Booster Sessions* were aimed at encouraging the participants to continually execute the action plan. Four strategies to strengthen the participants’ self-efficacy and motivation were used in all sessions in this phase: 1) obtaining performance accomplishments by experiencing success in achieving goals; 2) generating social persuasion through regular, sensible feedback and encouragement by the BCE facilitator; 3) gaining vicarious experiences through peer sharing, and 4) perceiving the positive physiological and emotional responses of engaging in regular exercise [32, 34].

The BCE programme consisted of six face-to-face one-hour sessions plus two booster sessions. The first three weekly sessions were arranged during the ‘goal initiation’ and ‘plan formulation’ phases. The remaining three sessions and two booster sessions were offered once per month in weeks 4, 8, and 12 during the programme; and at 2 and 6 months after the completion of the programme during the ‘action execution’ phase.

### Exercise training in the COMB and the EXER groups

A weekly, 45–60 min, centre-based exercise training programme that started from weeks 4 to 16 during the execution phase was provided to both the COMB and the EXER groups. The exercise training programme was designed based on the American Heart Association’s recommendations on exercise for older people [35]. All participants received circuit training with set exercises, which included balance, resistance, and aerobic exercises, but the dosage of the different components was tailor-made for each participant based on his/her physical condition. To prescribe an appropriate exercise dosage, the physiotherapist assessed each participant’s physical condition in the first exercise training session, and subsequently conducted monthly assessments. The dosage of exercises was gradually increased by increasing the number of repetitions and the duration of the exercises, and by using progressively heavier resistance bands based on the participants’ progress. An online video and a pamphlet describing the different types of exercises used in this programme was disseminated to all participants to encourage them to continually practise their exercises at home for approximately [36] minutes at least three times per week.

### Health talks for the EXER and control groups

Participants in the EXER and control groups attended centre-based health talks on managing different health issues, such as preventing falls and maintaining a healthy diet, but not on managing fatigue, in order to avoid contaminating the treatment effects of the COMB and EXER groups.

### Implementation procedures

The BCE programme was conducted by a well-trained facilitator with a master’s degree in psychology, who had received theoretical training related to BCEs. Before implementing the BCE programme, the facilitator completed training in BCE strategies provided by JYWL (the first author) who has successfully conducted psychosocial intervention trials to enhance behavioural changes in older adults with different health problems [36, 37]. A physiotherapist with rich experience in supervising exercise training for frail older people conducted the exercise programme. Another research assistant (RA) with a master’s degree in psychology and five years of experience working in clinical trials, who was not involved in other procedures of this study, ran the health talks. All face-to-face sessions had between 8 and 10 participants to maintain good interactions with the exercise instructor / BCE facilitator/ health talk speakers.

Some precautionary measures were taken during the COVID-19 pandemic. For instance, some post-intervention assessments and booster sessions at T2 and T3 were postponed during lockdown. In order to arrange interviews while ensuring the participants’ comfort, the research team contacted the centres to request use of private interview rooms, which allowed for one-on-one assessments to be made in a setting less likely to induce a fear of being too physically close to other people. As some participants were reluctant to undergo face-to face assessments due to health concerns, they were assessed by phone, so that it was not possible to conduct some assessments of their physical ability. From June to July 2020, offices and many venues in Hong Kong were allowed to resume operations at limited capacity. We therefore continued to conduct the T2 assessment (6 months post-intervention) as usual. As with the T2 assessment, the T3 assessment (12 months post-intervention), which took place in December 2020, proceeded normally because the centres had begun to resume operations.

### Outcome measures

Individual participants in all three groups were assessed on a variety of outcomes (described below) immediately after the 16-week intervention (T1), and at 6 months (T2), and 12 months (T3) post-intervention. The outcomes were compared with the baseline assessment (T0). The baseline assessment (T0) was conducted beginning in March 2018, while the 12-month post-intervention (T3) ended in December 2020.

### Primary outcome measures

The self-perceived levels of fatigue of each participant were assessed using the 20-item Chinese Multidimensional Fatigue Inventory (CMFI-20) [30, 38]. Each item was rated on a 5-point Likert scale, with a total score obtained by summing up all the items and ranging from 20 to 100, with higher scores indicating a higher level of fatigue. This scale was validated among 385 local cancer patients. A factor analysis revealed that the scale contains three factors (namely, physical, mental, and spiritual) with a factor loading ranging from 0.52 to 0.75. The Cronbach’s α was between 0.7 and 0.8 for the scores for the three domains and for the total of the CMFI-20 score [38]. This supports the view that the CMFI-20 is a reliable and valid instrument.

### Secondary outcomes measures

Physical endurance was evaluated using different physical tests. First, the participants’ upper- and low-limb strength was assessed using handgrip strength and the 30-s chair stand test [39]. Their mobility and overall physical endurance were assessed through the TUG test [27] and by their gait speed (i.e., the 2-min walk test) [37]. Handgrip strength was measured using a handheld dynamometer (Jamar Hydraulic Hand Dynamometer). The participants sat in a straight-backed chair with their feet flat on the floor and held the dynamometer in their dominant hand with their upper arm in line with their forearm at an approximately 90° angle, and with their wrist straight and unrotated. The average of the two readings taken was used for analysis. The 30-s chair stand test involved having the participants go from sitting to standing as many times as possible within 30 s, with higher scores indicating more instances of sitting/standing during a test interval and better lower limb muscle power. Depending on age and sex, older people can be at risk of falls if they are unable to perform a certain number of completions of sit to stand, ranging from less than 4 (for women aged 90–94 years) to 14 (for men in their 60s) [40]. The TUG measures walking speed by taking the time (in seconds) spent by the participants to execute a series of walking tasks, including standing up from a chair, walking for three metres, turning around, walking back to the chair, and sitting down [27]. The longer the time taken to complete the test, the greater the indication of poor functional mobility. Similar to the 6-Minute Walk Test (6MWT) and the 12-Minute Walk Test (12MWT), the 2MWT measures the self-paced walking ability of the participants, and it is particularly suitable for frail older people who cannot manage the longer walking tests [41]. In various studies, the 2MWT correlated highly with both the 6MWT and 12MWT, indicating that all three walking tests are similar in their ability to measure gait speed and exercise tolerance [40–42]

Exercise Self-efficacy, which refers to the participants’ self-confidence in their ability to exercise in a variety of circumstances (e.g., when feeling tired), was assessed using the 9-item Chinese Self-Efficacy for Exercise Scale (CSEE) [30, 34, 43]. Each item was rated on an 11-point Likert scale from 0 (i.e., no confidence) to 10 (i.e., most confidence). The final score is the sum of the ratings for all items, ranging from 0 to 90, with a higher score indicating more confidence. The CSEE was validated on 192 Chinese older people. Discriminant validity was shown by the CSEE total score, which significantly differentiated between individuals with or without regular exercise. The Cronbach’s α was 0.75, which showed an acceptable level of internal consistency [34].

Physical activity level was assessed using the 10-item Chinese Version of the Physical Activity Scale for the Elderly (PASE-C) [44] to measure self-reported occupational, household, and leisure activities for the last week. Its total score is calculated by multiplying the amount of time spent on each activity (hours/week) by the weight of the pre-set item. PASE-C was validated among 20 community-dwelling adults. The intraclass correlation of the scale was 0.704, and the Spearman’s rank correlation coefficient for total scores of the scale was 0.65 against the total accelerometer counts. This supports the view that the PASE-C is a reliable and valid instrument [45].

Frailty status was assessed based on the FFI, including i) an unintentional loss of 10% of body weight in the past year; ii) exhaustion: by answering ‘Yes’ to either ‘I felt that everything I did was an effort’, or ‘I could not get going in the last week’; iii) a slow walk time: with an average walking speed in the lowest quintile stratified by median body height; iv) reduced grip strength: with maximal grip strength in the lowest quintile stratified by body mass index quartile; and v) the PASE-C score in the lowest quintile (i.e., < 30 for men and < 27.7 for women) [44].

Depressive mood exhibited by the participants was assessed using the 15-item Chinese Geriatric Depression Scale (C-GDS) [46]. Each item was rated on a dichotomous scale from 0 to 1, and each negative answer was given 1 point, with a possible total score ranging from 0 to 15. The scores' 0 to 4', '5 to 9', and '10 to 15' indicate 'normal', 'mild', and 'moderate to severe' depressive mood, respectively. The C-GDS is commonly used in both clinical and research settings, and has shown good reliability and validity [46, 47].

### Sample size

The total summed score for fatigue, as measured by CMFI-20, was between 20 to 100. The changes in the mean fatigue scores were -0.58, -5.16, and -2.66 for the control, EXER, and COMB groups respectively, from pre-test to immediately post-intervention in the pilot study. The aim was to evaluate the preliminary effects of the same combined interventions in 79 frail older people with fatigue and a mean age of 79.32 (± 7.72) [30]. The Cohen’s d effect sizes for the EXER and COMB groups were 0.51 and 0.23, respectively; whereas the overall effect size was 0.21. As no similar cluster-RCT has been reported in the literature and the cause of general fatigue is heterogeneous, we assumed that an intra-correlation coefficient would be low (0.01) within each cluster [48]. Based on an effect size of 0.21 in the pilot study, and four clusters (community centres) per group with an intraclass correlation of 0.01, a significance level (α) of 0.05, and a power (1-β) of 0.8 for a two-sided test, the sample size was calculated to be 76 participants per group according to PASS 2021 [49]. For a long-term follow-up of 12 months, the plan was to recruit 285 participants (95 per group) after considering an attrition rate of 20%.

### Randomisation and allocation

Using computer-generated random numbers, a biostatistician not affiliated with this study randomised the centres into either: the COMB (i.e., the experimental), the active control (i.e., the EXER), and the passive control (i.e., the control) groups at a 1:1:1 ratio. Convenience sampling was used to recruit study participants from each centre. They were allocated to either the COME, the EXER, or the control group based on the randomised group allocated to their centre. To avoid selection biases, the allocation to the study groups was concealed from the researchers until the recruitment of the sample and measurement of the baseline had been completed.

### Blinding

Owing to the nature of the interventions used in this study, it was impossible to blind the participants and facilitators. Thus, only the outcome assessor was blinded to the group allocation of the centres and the participants.

### Statistical analysis

The data were analysed using the statistical package SPSS 27 (IBM Corp, Armonk, NY) for Windows. Missing data were handled by using multiple imputation, which involved identifying the missing data and replacing them with multiple plausible values based on the available data to achieve an intention-to-treat analysis. Descriptive statistics were generated for the demographic data. Normality assumptions for the variables were checked. The baseline characteristics of the participants in the three groups were compared using ANOVA for the continuous variables, a chi-square test for the categorical variables, and the Kruskal–Wallis test for the ordinal variables. A generalised estimating equation (GEE) adjusted for age, because a significant difference in age was identified among the three groups, was used to examine the group effect, time effect, and their interaction in the outcome assessments across the three post-tests (immediately [T1], 6 months [T2], and 12 months [T3] after the intervention) among the three groups. As multiple comparisons were carried out (i.e., T0-T1, T0-T2, T0-T3), a *p*-value of < 0.017 (0.05/3) was considered statistically significant based on a Bonferroni adjustment [50].

## Results

### Characteristics of the participants

**Fig. 2:**
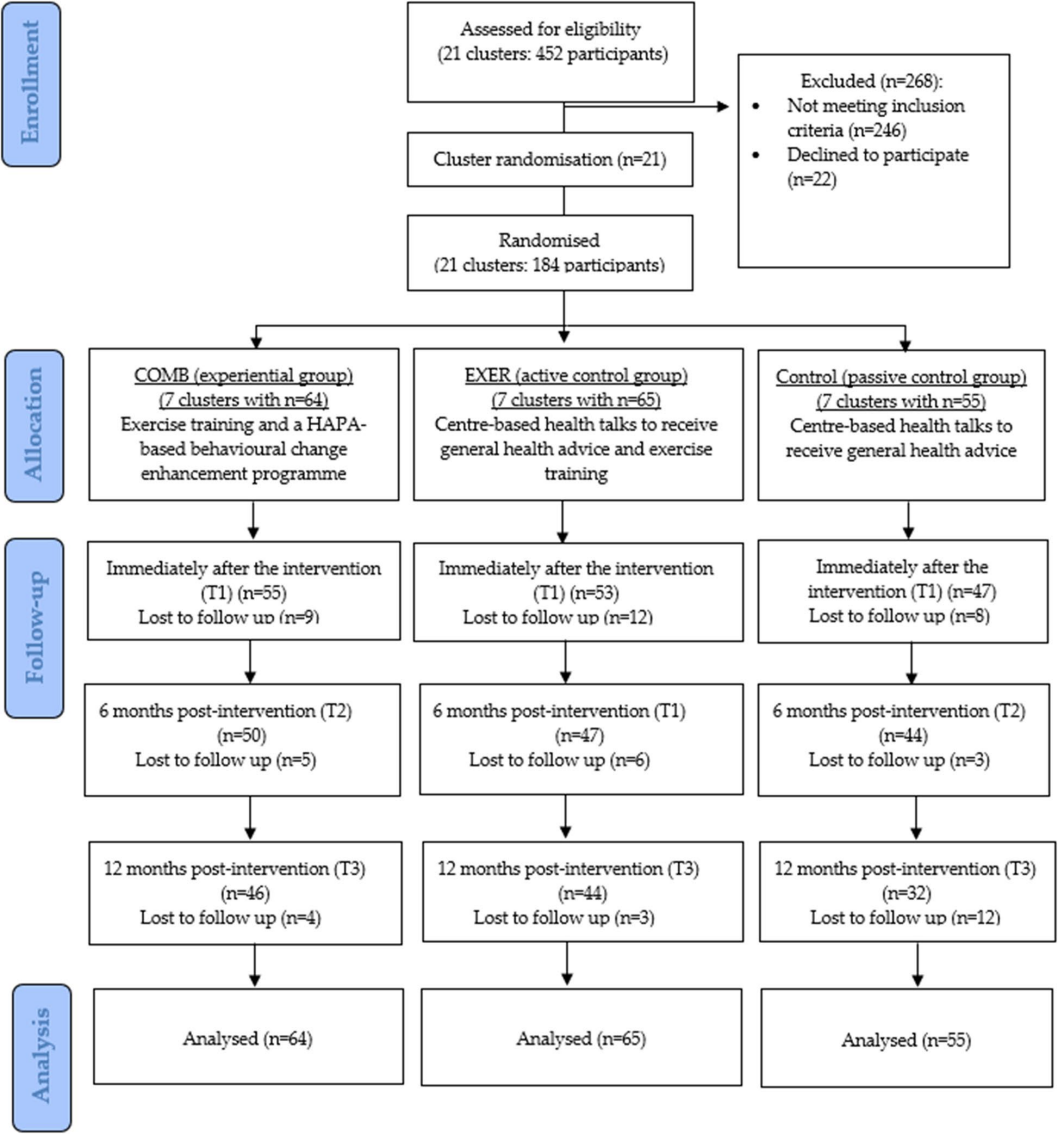
CONSORT flow diagram of the subject recruitment and study procedure

The recruitment period started during the period of social unrest that began in June 2019. The turmoil continued with the outbreak of the COVID-19 pandemic in the beginning of 2020. The resulting social distancing/ quarantine policy and lockdowns acted as a severe drag on the project, and individuals expressed concerns about participating in the study. A total of 184 participants from 21 community centres were recruited between January 2018 and April 2020 and allocated into either the COMB, EXER, or control groups (COMB: *n* = 64, EXER: *n* = 65, control: *n* = 55) based on cluster randomisation at a ratio of 1:1:1. Both situations had made recruitment and retention of participants far more difficult than would otherwise have been the case. Therefore, the sample size was only about 64.6% of the sample size calculated in the proposal. The attrition rates were 14.1%, 18.5%, and 14.5% at T1 (immediately post-intervention), increasing to 28.1%, 32.3%, and 41.8% at T3 (at the 12-month follow-up) in the COMB, EXER, and control groups (see Fig. 2). The attrition rates of the participants at T3 were higher than the estimated attrition rate of 20% in the calculation of sample size.

The demographic and clinical characteristics of the participants are summarised in Table 1. About 90% were female (*n* = 165) and their mean age was 79.1 (SD = 6.4). More than half of the participants (*N* = 105, 57%) reported not using any walking aids. The result of the chi-square test and one-way ANOVA revealed no group differences in demographic and baseline measurements among the three arms, with the exception of a significant difference in age.

**Table 1:** Characteristics of the study participants

	**All**	**COMB**	**EXER**	**CONTROL**	**Statistics**^a^	***P***
	**(*n* = 184)**	**(*n* = 64)**	**(*n* = 65)**	**(*n* = 55)**		
**Age (years)-Mean (SD)**	79.1 (6.4)	78.7 (6.5)	77.9 (5.8)	81.1 (6.7)	F = 3.925	0.021
**Gender N (%)**						
Male	19 (10.3)	10 (15.6)	3 (4.6)	6 (10.9)	X^2^ = 4.250	0.119
Female	165 (89.7)	54 (84.4)	62 (95.4)	49 (89.1)		
**Walking aids N (%)**					X^2^ = 7.145	0.848
None	105 (57.4)	40 (62.5)	36 (55.4)	29 (53.7)		
Walking stick	45 (24.6)	15 (23.4)	16 (24.6)	14 (25.9)		
Crutches	22 (12.0)	6 (9.3)	9 (13.8)	7 (12.9)		
Walking frames	2 (1.1)	0 (0)	0 (0)	2 (3.7)		
Rollators	2 (1.1)	1 (1.6)	1 (1.5)	0 (0)		
Shopping trolley	3 (1.6)	1 (1.6)	1 (1.5)	1 (1.9)		
Others	4 (2.2)	1 (1.6)	2 (3.2)	1 (1.9)		

^a^ Either Chi-square test or one-way ANOVA was used depending on the types of dependent variables

The baseline outcome data of all of the 184 participants are summarised in Table 2. For baseline outcomes, the result of the one-way ANOVA suggested that there were no statistically significant differences between the three groups in any of the primary and secondary outcomes, indicating that the participants in the three groups were similar in terms of level of fatigue, physical endurance, exercise self-efficacy, physical activity level, and psychological wellbeing.

**Table 2:** Outcome variables at baseline^a^

	**TOTAL**	**COMB**	**EXER**	**CONTROL**	Statistics^*^	***p*-value**
	**(*n* = 184)**	**(*n* = 64)**	**(*n* = 65)**	**(*n* = 55)**		
	**Mean (SD)**	**Mean (SD)**	**Mean (SD)**	**Mean (SD)**		
**Primary outcome**						
Multi-dimensional fatigue inventory (CMFI-20), 20–100, higher = more fatigue	66.4 (13.2)	69.3 (13.9)	64.3 (13.3)	65.4 (11.9)	F = 2.549	*p* = 0.081
**Secondary outcomes**						
***Physical endurance***						
Hand Grip Strength	11.4 (5.6)	11.8 (6.4)	11.5 (4.8)	10.8 (5.6)	F = 0.541	*P* = 0.583
30-s Chair Stand Test	8.2 (3.7)	8.1 (3.5)	8.7 (3.4)	7.6 (4.1)	F = 1.434	*p* = 0.241
Timed-Up-and-Go test (TUG)	15.6 (7.9)	15.4 (6.6)	14.4 (5.4)	17.2 (11.1)	F = 1.951	*p* = 0.145
2-min Walk Test	73.6 (27.0)	73.9 (30.2)	74.0 (25.2)	72.5 (25.6)	F = 0.057	*p* = 0.944
***Exercise and activity level related***						
Exercise Self-efficacy Scale (CSEE), 0 -90, higher = higher self-efficacy	39.7 (17.9)	39.1 (16.1)	40.6 (18.8)	39.1 (18.9)	F = 0.151	*p* = 0.860
^ #^PASE-C, 0–400, higher = more physically active	48.6 (25.4)	49.4 (24.9)	51.4 (23.8)	44.5 (27.7)	F = 1.151	*p* = 0.319
***Frailty related***						
Fried frailty index (FFI), 0–5, higher = frailer	2.8 (0.8)	2.8 (0.8)	2.6 (0.8)	2.9 (0.7)	F = 2.024	*p* = 0.135
***Psychological wellbeing related***						
Geriatric depression scale (C-GDS), 0–15, > 8 = depression	4.9 (3.7)	5.5 (3.7)	4.5 (3.5)	4.8 (3.9)	F = 1.213	*p* = 0.300

^#^PASE−C: The Chinese Version of the Physical Activity Scale for the Elderly

^*^One−way ANOVA was used to assess group differences in outcome variables at baseline

^a^adjusted for age

### Treatment effects of the intervention on the primary outcome

The fatigue scores (CMFI-20) by group (COMB, EXER, control) over time (T0, T1, T2, T3) are shown in Table 3. The GEE analyses showed that there was a significant interaction effect (time x group) between the COMB and the control groups immediately post-intervention (T0 vs T1: Beta: -7.59, SE = 2.19, *p* < 0.001), and at 6 months post-intervention (T0 vs T2: Beta: -8.12, SE = 2.28, *p* < 0.001) and 12 months post-intervention (T0 vs T3: Beta: -11.39, SE = 2.45, *p* < 0.001). Similarly, when comparing the COMB to the EXER groups (active control), there was a significant interaction effect immediately post-intervention and at 12 months post-intervention (T0 vs T1: Beta: -5.71, SE = 2.31, *p* = 0.013; T0 vs T3: Beta: -6.59, SE = 2.44, *p* = 0.007). However, no significant differences were noted between the EXER group and the control group at any of the time points.

**Table 3:** Results of the GEE Analyses on all outcome measures^a^

	**COMB Mean (SD)**	**EXE Mean (SD)**	**CTRL Mean (SD)**	**Time effect**		**Group effect**						**Time x Group effect**					
						**COMB vs CTRL**		**EXE vs CTRL**		**COMB vs EXE**		**COMB vs CTRL**		**EXE vs CTRL**		**COMB vs EXE**	
				**Beta (SE)**	***p***	**Beta (SE)**	***p***	**Beta (SE)**	***p***	**Beta (SE)**	***p***	**Beta (SE)**	***p***	**Beta (SE)**	***p***	**Beta (SE)**	***p***
**Primary Outcome**																	
**CMFI-20, 20–100,** **higher = more fatigue**				-	-	3.84 (2.35)	0.10	-1.16 (2.28)	0.61	5.00^*^ (2.37)	0.04	-	-	-	-	-	-
T0	69.3 (13.90)	64.3 (13.30)	65.4 (11.90)			-	-	-	-	-	-	-	-	-	-	-	-
T1	62.3 (14.17)	62.4 (14.21)	66.4 (12.60)	0.48 (1.27)	0.70	-	-	-	-	-	-	-7.59^***^ (2.19)	< 0.001	-1.82 (1.94)	0.35	-5.71^*^ (2.31)	0.01
T2	64.28 (12.69)	63.6 (13.54)	67.3 (11.69)	2.43 (1.43)	0.09	-	-	-	-	-	-	-8.12^***^ (2.28)	< 0.001	-2.70 (2.24)	0.23	-5.36^*^ (2.48)	0.03
T3	61.1 (11.33)	62.7 (14.21)	69.7 (12.49)	4.08^*^ (1.60)	0.01	-	-	-	-	-	-	-11.39^***^ (2.45)	< 0.001	-4.76^*^ (2.25)	0.03	-6.59^*^ (2.44)	0.01
**Secondary outcome**																	
**Handgrip Strength**				-	-	1.07 (1.09)	0.33	0.70 (0.96)	0.46	0.37 (0.99)	0.71	-	-	-	-	-	-
T0	11.8 (6.42)	11.5 (4.82)	10.8 (5.62)	-	-	-	-	-	-	-	-	-	-	-	-	-	-
T1	13.8 (6.93)	10.3 (5.69)	10.1 (5.16)	-1.07* (0.52)	0.04	-	-	-	-	-	-	2.91^***^ (0.85)	< 0.001	-0.47 (0.93)	0.61	3.39^***^ (1.03)	< 0.001
T2	9.7 (5.65)	12.2 (5.93)	10.5 (5.76)	-0.10 (0.84)	0.91	-	-	-	-	-	-	-1.38 (1.11)	0.21	0.19 (1.12)	0.86	-1.55 (1.04)	0.14
T3	13.7 (7.09)	15.0 (4.72)	10.4 (5.36)	-0.29 (0.89)	0.75	-	-	-	-	-	-	1.08 (1.12)	0.34	4.09^***^ (1.11)	< 0.001	-3.02^***^ (0.95)	< 0.001
**2-min walk test**				-	-	2.89 (5.16)	0.58	2.99 (4.71)	0.53	-0.10 (4.86)	0.98	-	-	-	-	-	-
T0	73.9 (30.20)	74.0 (25.20)	72.5 (25.60)	-	-	-	-	-	-	-	-	-	-	-	-	-	-
T1	87.4 (29.55)	77.4 (20.75)	68.4 (28.03)	-3.22 (2.38)	0.18	-	-	-	-	-	-	16.57^***^ (4.18)	< 0.001	8.85^*^ (3.62)	0.01	7.70 (4.38)	0.079
T2	74.8 (29.72)	74.0 (26.69)	57.7 (28.52)	-7.38 (5.54)	0.18	-	-	-	-	-	-	15.72^*^ (6.96)	0.02	11.47 (6.68)	0.09	4.28 (5.63)	0.447
T3	83.1 (26.73)	79.5 (22.87)	66.5 (27.13)	-3.42 (2.82)	0.22	-	-	-	-	-	-	7.24 (5.05)	0.15	7.97^*^ (3.83)	0.04	-0.78 (4.94)	0.875
**TUG**						-0.11 (0.10)	0.28	-0.18 (0.10)	0.07	0.07 (0.07)	0.31	-	-	-	-	-	-
T0	15.4 (6.55)	14.4 (5.40)	17.2 (11.06)			-	-	-	-	-	-	-	-	-	-	-	-
T1	12.5 (6.31)	12.2 (4.32)	15.5 (6.93)	-0.07 (0.06)	0.28	-	-	-	-	-	-	-0.17 (0.09)	0.06	-0.07 (0.07)	0.37	-0.09 (0.07)	0.21
T2	13.0 (4.43)	13.6 (5.68)	17.7 (8.43)	-0.04 (0.06)	0.50	-	-	-	-	-	-	-0.22^*^ (0.09)	0.015	-0.10 (0.08)	0.25	-0.13 (0.09)	0.170
T3	12.8 (5.26)	12.4 (4.38)	17.5 (9.02)	-0.01 (0.07)	0.88	-	-	-	-	-	-	-0.11 (0.09)	0.206	-0.12 (0.07)	0.10	0.01 (0.07)	0.94
**30-s chair stand test**						0.41 (0.70)	0.56	1.04 (0.69)	0.14	-0.63 (0.62)	0.31	-	-	-	-	-	-
T0	8.0 (3.53)	8.7 (3.38)	7.6 (4.10)	-	-	-	-	-	-	-	-	-	-	-	-	-	-
T1	10.3 (3.29)	9.3 (3.32)	8.2 (4.57)	0.51 (0.49)	0.30	-	-	-	-	-	-	1.76^**^ (0.62)	0.004	0.14 (0.68)	0.84	1.62^**^ (0.61)	0.007
T2	10.2 (4.34)	9.2 (3.90)	7.9 (4.54)	0.16 (0.71)	0.82	-	-	-	-	-	-	2.33^*^ (1.00)	0.02	0.91 (0.89)	0.31	1.43 (0.89)	0.11
T3	10.3 (4.25)	10.3 (3.36)	7.6 (4.87)	-0.21 (0.53)	0.69	-	-	-	-	-	-	2.17^**^ (0.80)	0.007	1.49 (0.71)	0.04	0.67 (0.77)	0.38
**CESS,** **0 -90,** **higher = higher** **self-efficacy**						0.00 (3.22)	1.00	1.52 (3.42)	0.66	-1.52 (3.05)	0.62	-	-	-	-	-	-
T0	39.1 (16.11)	40.6 (18.76)	39.1 (18.93)	-	-	-	-	-	-	-	-	-	-	-	-	-	-
T1	46.1 (17.86)	47.5 (17.38)	34.4 (19.18)	-4.78 (2.55)	0.06	-	-	-	-	-	-	12.03^***^ (3.65)	< 0.001	10.31^**^ (3.65)	0.005	1.93 (3.69)	0.60
T2	40.8 (16.62)	34.3 (16.12)	34.7 (18.91)	-6.13 (4.59)	0.18	-	-	-	-	-	-	7.47 (5.82)	0.20	1.82 (5.54)	0.74	5.52 (4.7)	0.25
T3	42.2 (18.89)	47.8 (22.75)	34.7 (23.93)	-4.40 (5.02)	0.38	-	-	-	-	-	-	7.07 (5.72)	0.22	10.97 (6.03)	0.07	-3.85 (4.34)	0.38
**PASE-C, 0–400, higher = more physically active**						4.90 (4.83)	0.31	6.93 (4.72)	0.14	-2.03 (4.26)	0.63	-	-	-	-	-	-
T0	49.4 (24.91)	51.4 (23.78)	44.5 (27.75)	-	-	-	-	-	-	-	-	-	-	-	-	-	-
T1	83.5 (37.88)	80.1 (31.58)	48.3 (26.01)	3.92 (5.02)	0.43	-	-	-	-	-	-	29.89^***^ (7.32)	< 0.001	23.54^***^ (6.74)	< 0.001	6.42 (6.97)	0.36
T2	64.5 (35.23)	50.6 (26.78)	42.3 (34.35)	-1.17 (7.40)	0.87	-	-	-	-	-	-	17.53 (10.02)	0.08	1.35 (9.22)	0.88	16.20 (8.70)	0.06
T3	62.2 (34.79)	74.6 (36.84)	41.2 (22.64)	-4.34 (4.37)	0.32	-	-	-	-	-	-	15.67^*^ (6.84)	0.02	27.17^***^ (6.97)	< 0.001	-11.60 (7.56)	0.13
**FFI, 0–5, high = frailer**						-0.13 (0.13)	0.34	-0.28^*^ (0.13)	0.04	0.15 (0.14)	0.27	-	-				
T0	2.8 (0.77)	2.6 (0.78)	2.9 (0.69)	-	-	-	-	-	-	-	-	-	-	-	-	-	-
T1	1.8 (0.78)	1.8 (0.68)	2.6 (0.83)	-0.25^*^ (0.13)	0.05	-	-	-	-	-	-	-0.68^***^ (0.17)	< 0.001	-0.54^**^ (0.19)	0.005	-0.14 (0.18)	0.46
T2	2.1 (0.87)	2.1 (1.05)	2.7 (0.97)	-0.35 (0.19)	0.07	-	-	-	-	-	-	-0.47 (0.26)	0.073	-0.26 (0.27)	0.34	-0.21 (0.27)	0.44
T3	1.7 (0.89)	1.7 (0.79)	2.7 (0.82)	-0.21 (0.11)	0.06	-	-	-	-	-	-	-0.73^***^ (0.21)	< 0.001	-0.64^***^ (0.19)	< 0.001	-0.10 (0.23)	0.66
**C-GDS, 0–15, > 8 = depression**			-			0.68 (0.69)	0.33	-0.31 (0.68)	0.64	0.99 (0.63)	0.11	-	-	-	-	-	-
T0	5.5 (3.66)	4.5 (3.51)	4.8 (3.91)	-	-	-	-	-	-	-	-	-	-	-	-	-	-
T1	4.9 (3.57)	5.1 (3.78)	5.5 (3.91)	0.55 (0.38)	0.15	-	-	-	-	-	-	-1.24 (0.64)	0.06	0.10 (0.54)	0.86	-1.32^*^ (0.64)	0.04
T2	6.2 (4.28)	4.9 (2.85)	5.2 (4.27)	0.65 (0.58)	0.26	-	-	-	-	-	-	-0.06 (0.86)	0.94	-0.56 (0.70)	0.42	0.50 (0.76)	0.510
T3	4.2 (3.55)	5.2 (3.82)	6.6 (4.00)	1.64^**^ (0.57)	0.004	-	-	-	-	-	-	-2.76^***^ (0.78)	< 0.001	-0.85 (0.76)	0.26	-1.91^*^ (0.73)	0.01

*CTRL* Control group, *FFI* Fried Frailty Index, *CGDS* Geriatric depression scale, *PASE* Physical activity scale for the elderly, *TUG* Timed-Up-and-Go test, *CESS* Chinese Self-Efficacy for Exercise Scale

T0: baseline, T1: immediately post-intervention, T2: 6-month follow-up, T3: 12- month follow-up;

^a^ adjusted for age, a logarithmic transformation was conducted for the GEE analysis

^*^p < 0.05

^**^p < 0.01

^***^p < 0.001

### Treatment effects of the intervention on the secondary outcomes

#### Effects on physical functioning

The physical endurance of the participants was assessed by the change in their scores on handgrip strength, the 30-s chair stand test, TUG, and the 2-min walk test. For handgrip strength, significant interaction effects between the COMB and the EXER groups, as well as between the COMB and the control groups, were observed post-intervention (T0 vs T1, COMB vs Control: Beta = 2.91, SE = 0.85, *p* < 0.001; T0 vs T1, COMB VS EXER: Beta = 3.39, SE = 1.03, *p* < 0.001). A significant interaction effect on handgrip strength was also observed between the EXER and control groups at the 12-month follow-up (T0 vs T3: Beta = 4.09, SE = 1.11, *p* < 0.001) as well as between the COMB and EXER groups (T0 vs T3: Beta = -3.02, SE = 0.95, *p* < 0.001). For the 30-s chair stand test, there was a significant interaction effect between the COMB and the control groups immediately post-intervention and at 12 months post-intervention (T0 vs T1: Beta = 1.76, SE = 0.62, *p* = 0.004; T0 vs T3: Beta = 2.17, SE = 0.80, *p* = 0.007). When comparing the COMB and the EXER groups, a significant interaction effect could only be found immediately post-intervention (T0 vs T1: Beta = 1.62, SE = 0.61, *p* = 0.007). However, there were no significant differences between the EXER group and the control group at any time point.

For the TUG test, a significant interaction effect between the COMB and the control groups at 6 months post-intervention (T0 vs T2: Beta = -0.022, SE = 0.09, *p* = 0.015) was identified, but not at other time points or when the COMB was compared with the EXER groups. For the 2-min walk test, the results suggested that there was a significant interaction effect between the COMB and the control groups at post-intervention (T0 vs T1: Beta = 16.57, SE = 4.18, *p* < 0.001) as well as between the EXER and the control groups (T0 vs T1: Beta = 8.85, SE = 3.62, *p* = 0.01), but not at any follow-up time points. There were no significant interaction effects at any time point when comparing the COMB group with the EXER group.

### Effects on physical activity level and self-efficacy in exercise

With regard to the exercise self-efficacy score, a significant interaction effect was only observed at post-intervention when the COMB was compared to the control (T0 vs T1: Beta = 12.03, SE = 3.65, *p* < 0.001), as well as when the EXER was compared to the control (T0 vs T1: Beta = 10.31, SE = 3.65, *p* = 0.01).

For PASE-C, which reflected the participants’ physical activity levels, there was a significant interaction effect between the COMB and the control at post-intervention (T0 vs T1: Beta = 29.89, SE = 7.32, *p* < 0.001). There was a significant interaction effect between the EXER and the control at post-intervention (T0 vs T1: Beta = 23.54, SE = 6.74, *p* < 0.001) and at the 12-month follow-up (T0 vs T3: Beta = 27.17, SE = 6.97, *p* < 0.001. No interaction effect was observed at any follow-up time point when the COMB group was compared with the EXER group.

### Effect on frailty

With regard to the frailty score (assessed by FFI), our result suggested that there was a significant interaction effect between the COMB and the control groups at post-intervention (T0 vs T1: Beta = -0.68, SE = 0.17, *p* < 0.001) and at the 12-month follow-up (T0 vs T3: Beta = -0.73, SE = 0.21, *p* < 0.001), but not at the 6-month follow-up. A similar observation was made when the EXER and the control groups were compared – we found a significant interaction effect only at post-intervention and at the 12-month follow-up (T0 vs T1: Beta = -0.54, SE = 0.19, *p* = 0.01; T0 vs T3: Beta = -0.64, SE = 0.19, *p* < 0.001).

### Effect on psychological wellbeing

We assessed changes in psychological wellbeing using GDS. When comparing the COMB group with the control group, the results suggested that there were no interaction effects from the GDS score at post-intervention and at the 6-month follow-up, but that such an effect became significant at the 12-month follow-up (T0 vs T3: Beta = -2.76, SE = 0.78, *p* < 0.001). When comparing the COMB group with the EXER group, there was a significant interaction effect only at 12 months post-intervention (T0 vs T3: Beta = -1.91, SE = 0.73, *p* = 0.01).

## Discussion

The present trial showed that despite the higher fatigue score among participants in the COMB group at baseline, their scores dropped significantly across the study time points compared to the fatigue scores of the EXER and control groups. The COMB group also had statistically better immediate and sustained effects to 12 months on reducing aged-induced fatigue in frail older people compared with the EXER group that received exercise training or the control group that received the health talks. A greater improvement in psychological wellbeing was also observed in the COMB group than in the group that received exercise training and in the control group at the 12-month follow-up.

The COMB programme also had superior effects than the control group on increasing the participants’ physical endurance in terms of handgrip strength, and in functional mobility as measured by the 2-min walking test and the 30-s chair stand test immediately post-intervention. However, the effect of the COMB programme on all domains of physical functioning did not differ from that of the exercise programme alone, except with respect to upper limb strength immediately post-intervention and at the 12-month follow-up.

Both the COMB programme and exercise training alone had superior immediate effects on the participants’ exercise self-efficacy, physical activity levels, and frailty status compared with the control group. These significant positive effects on frailty status could be observed at the 12-month follow-up, but not at the 6-month follow-up. However, the effects of the COMB programme and exercise training alone on the participants’ exercise self-efficacy, physical activity levels, and frailty status were similar, as no significant differences between these two intervention groups could be found at any measurement time point.

The results of our study were consistent with those of previous intervention studies, which demonstrated that exercise training could improve physical endurance, physical activity levels, exercise self-efficacy, and reverse frailty status [51, 52]. However, exercise could impose a burden on participants and lead to exhaustion for those who consistently experienced a feeling of fatigue. This is because the energy generated during exercise may exhaust the energy stocks of the body, thereby causing the frail older adults to feel that they had exceeded their physical limits, leading to low motivation and self-efficacy [53]. This may explain why the exercise programme alone did not affect the participants’ fatigue levels. Its effects on the participants’ physical endurance were also inconsistent. Only in handgrip strength at the 12-month follow-up and in the 2-min walk immediately post-invention did participants who participated in the exercise perform significantly better than did the control group. Referring to the decondition model [54], older people with general fatigue may avoid engaging in exercise due to a fear that physical overexertion may induce muscle pain/weakness. This explains why older people frequently cite fatigue as a cause of their non-adherence to or withdrawal from exercise programmes [13]. Their inability to function at previous levels may lead to frustration and to low motivation and self-efficacy in their daily activities, thus becoming a major barrier to their regular engagement in exercise [55, 56].

In our study, the motivational phase of the BCE programme provided participants in the COMB with more psychological preparation and support to encourage them to do the exercises voluntarily instead of being forced. This was achieved by using three key attributes of the HAPA model (i.e., self-efficacy, outcome expectancies, and risk awareness) to enhance their motivation to manage their health problem [20]. In addition, the volitional phase of the BCE programme ensured that the participants would execute the exercise protocol by providing them with help to draw up make a detailed plan, sharing the successful experiences of peers, encouraging them to make achievements, and solving barriers that arose during the execution process. The empirical evidence shows that the HAPA model can be effectively used as a conceptual framework to design concrete strategies to motivate people to change their behaviour. Used alongside exercise interventions, these strategies can promote adherence to exercise regimes among those undergoing orthopaedic rehabilitation [22] and cardiac rehabilitation [21, 57], and among older patients with sedentary lifestyles [58]. These findings are similar to those from the current study using the BCE programme, which gave the participants a better experience when engaging in exercise. The strategies that were used were identified as being effective at improving the participants’ motivation and psychological wellbeing. Therefore, they were more willing to exercise, leading to other positive outcomes related to their physiological wellbeing.

The COMB programme used in our trial had better immediate and sustained results than the exercise training alone in reducing general fatigue. This could be because the BCE programme captured the core parts of the participants’ motivation and psychological changes. For older people, psychological wellbeing tends to be important for managing fatigue and symptoms of frailty [59]. Depressive mood may occur during the ageing process, leading to low motivation and self-efficacy. Depression is believed to be one of the important factors behind unexplained fatigue in older adults, leading to a decline in physical activity levels [14, 60]. Conversely, older people with fatigue were found to be more depressed than those without fatigue [61]. Therefore, to help this particular group of older adults overcome a complex phenomenon of fatigue that affects both their physical and psychological wellbeing, BEC strategies to enhance their motivation and self-efficacy are as crucial as providing them with physical exercise training.

The strengths of the present study include methodologically rigorous and long-term follow-ups. This study was designed and conducted strictly following the Consort statement for cluster randomised trials [23]. In addition, the one-year follow-up measurements provided preliminary evidence of the possible long-term effects of the combined intervention. The present study also shows the potential of this programme to be implemented in the community to prevent frailty in the early stage and to ease the healthcare burden on society.

This study has several limitations that warrant acknowledgement and consideration. Firstly, the actual number of participants was smaller than originally planned due to unforeseeable events, including the social unrest and the COVID-19 pandemic. The sample size was not reduced in the phase of sample size estimation, however the abovementioned incidents negatively affected the recruitment and retention of participants. Although the final sample size of 189 participants seems to have sufficient statistical power to determine changes in levels of fatigue among the three intervention groups at different outcome measurement time points (i.e., based on the GEE analyses on the fatigue scores, please refer to the results section), it seems insufficient for determining the effects on most of the secondary outcomes, particularly the effects between the COMB and the EXER groups. Secondly, the relatively high loss to follow-up, particularly in T3, may induce attrition bias. However, the independent t-test (supplementary information Tables 1 and 2) suggested that there were no statistically significant differences in any of the demographic data or in the primary and secondary outcomes between participants who completed the study versus those who withdrew from it. These findings can to some extent reduce the concern about attrition bias. Due to the relatively high attrition rate, readers should be cautious when interpreting the findings. Thirdly, significant differences in age were identified among the three interventional groups. However, adjustments were made for this variable in the statistical analysis. Fourthly, 90% of our participants were women, limiting the generalizability of our findings with respect to frail older men with fatigue. Fifthly, we did not explore the effects of disease-related fatigue, such as cardiac and pulmonary diseases, which are also common in older adults. Thus, the findings may not apply to managing disease-related fatigue. Lastly, we did not monitor the participants' daily adherence to the recommended exercise regimen. A wearable activity tracker should be used in a future study to monitor the participants’ daily activity levels to reflect their adherence to the exercise regimen.

## Conclusions

Ageing-induced fatigue often causes older adults to feel exhausted and debilitated, both physiologically and psychologically. Thus, an intervention with multiple components that can address both the physiological and psychological problems caused by fatigue is essential. It should be able enhance the participants’ motivation and self-efficacy to adhere to the exercise training, leading to other positive physiological and psychological outcomes. The combined intervention of the BCE programme and exercise used in this trial showed more significant immediate and sustainable benefits than exercise alone or health education in improving general fatigue in frail older adults, particularly, older women. This intervention has the potential to be sustainably implemented in the community to prevent frailty in the early stage and to help ease the healthcare burden on society. However, further research studies are still required to confirm its applicability.

## Supplementary Information

**Additional file 1.**

## Data Availability

The datasets used and/or analysed during the current study are available from the corresponding author on reasonable request.

## Abbreviations

BCE: Behaviour change enhancement
COMB: Exercise combined with behaviour change enhancement
EXER: Exercise
HAPA: Health action process approach
TUG: Timed-up-and-go test
