# Supplementary material for: Effects of an individualised exercise programme plus Behavioural Change Enhancement (BCE) strategies for managing fatigue in frail older adults: a cluster randomised controlled trial
Source: BMC Geriatr. 2023 Jun 16;23:370. doi: 10.1186/s12877-023-04080-0 (PMC10273765; doi:10.1186/s12877-023-04080-0)
Supplement: Supplementary file 1 — Additional file 1. [file 12877_2023_4080_MOESM1_ESM.pdf]

Supplementary information [see Additional file]

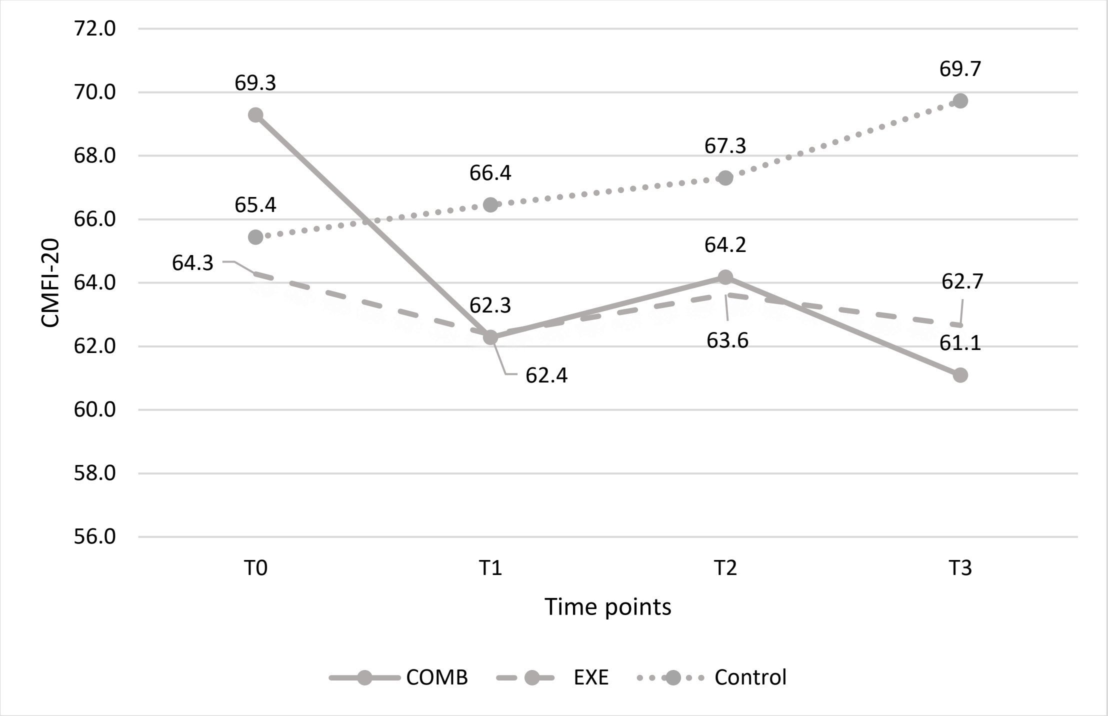

CMFI-20: Chinese Multidimensional Fatigue Inventory (CMFI-20)

Figure 3.1 Changes in CMFI-20 scores in the three study groups over time points.

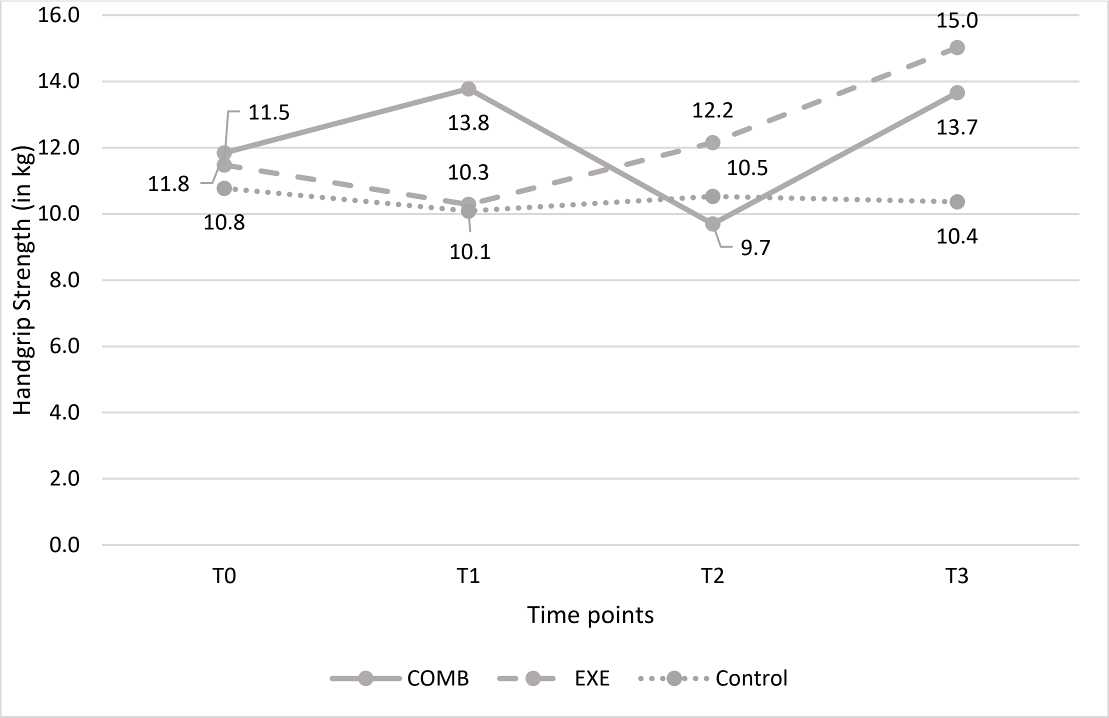

Figure 3.2 Changes in handgrip strength in the three study groups over time points.

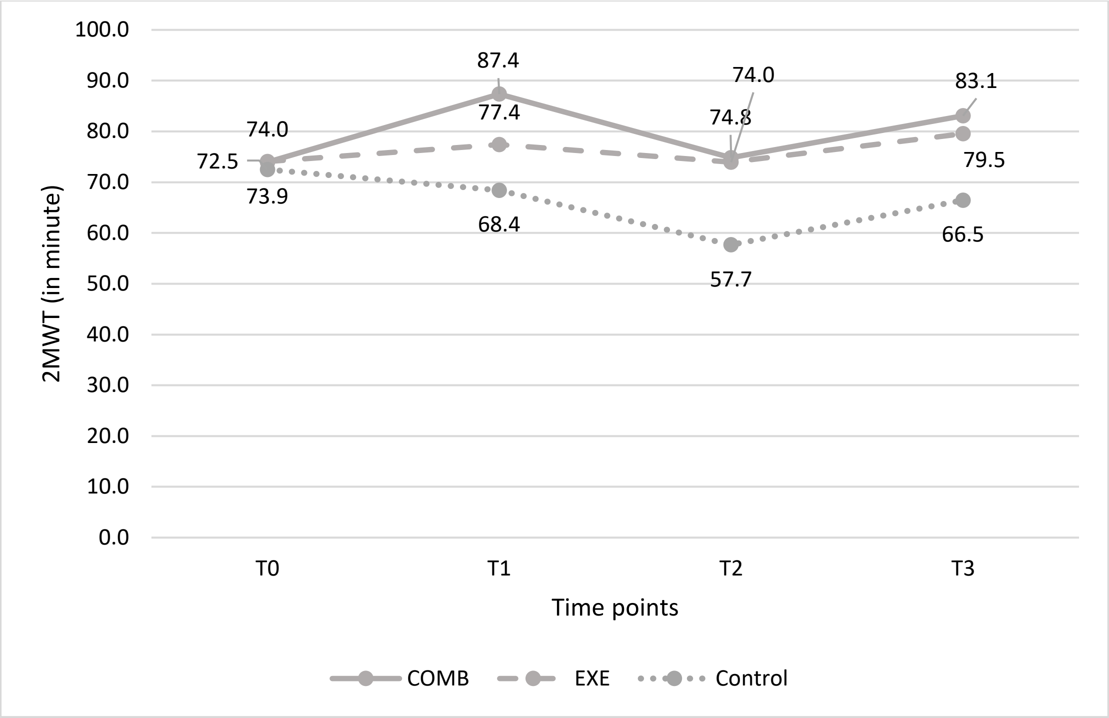

2MWT: 2-Minute Walk Test

Figure 3.3 Changes in 2MWT in the three study groups over time points.

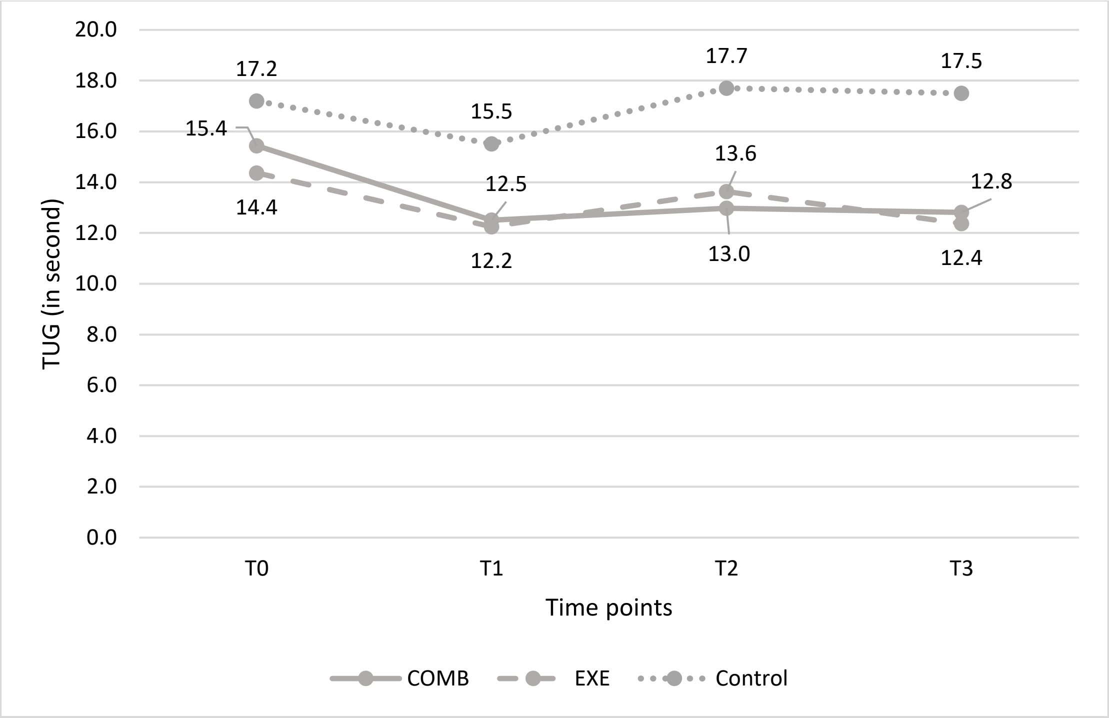

TUG: Timed Up and Go

Figure 3.4 Changes in the TUG test in the three study groups over time points.

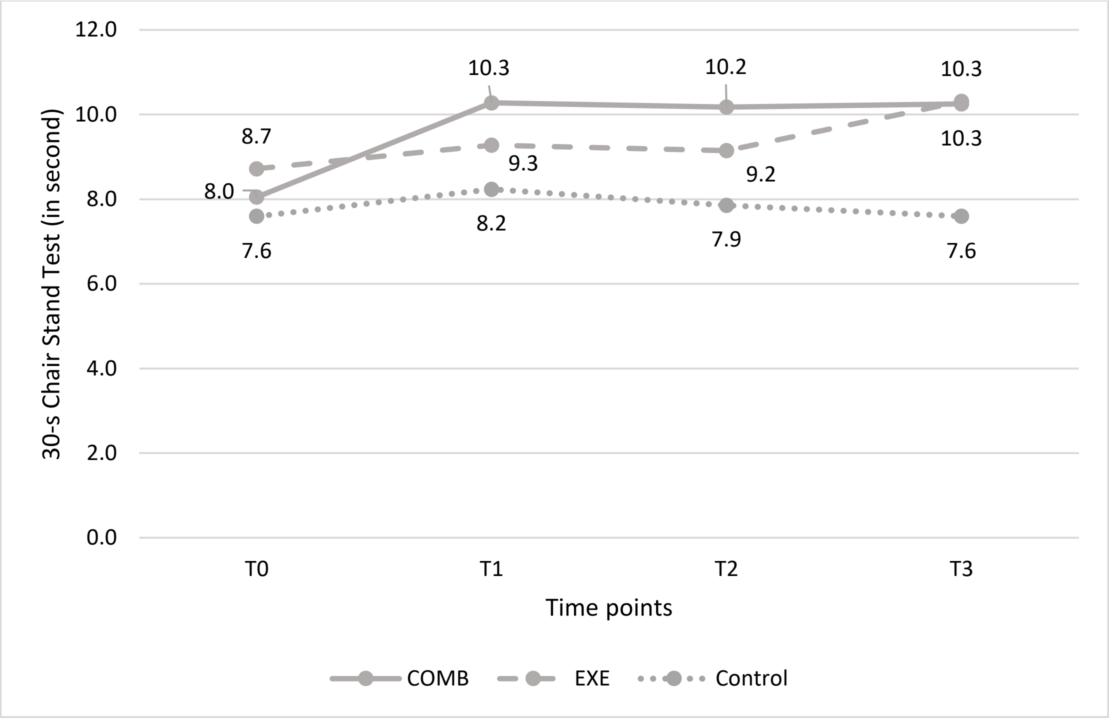

Figure 3.5 Changes in the 30-second Chair Stand Test in the three study groups over time points.

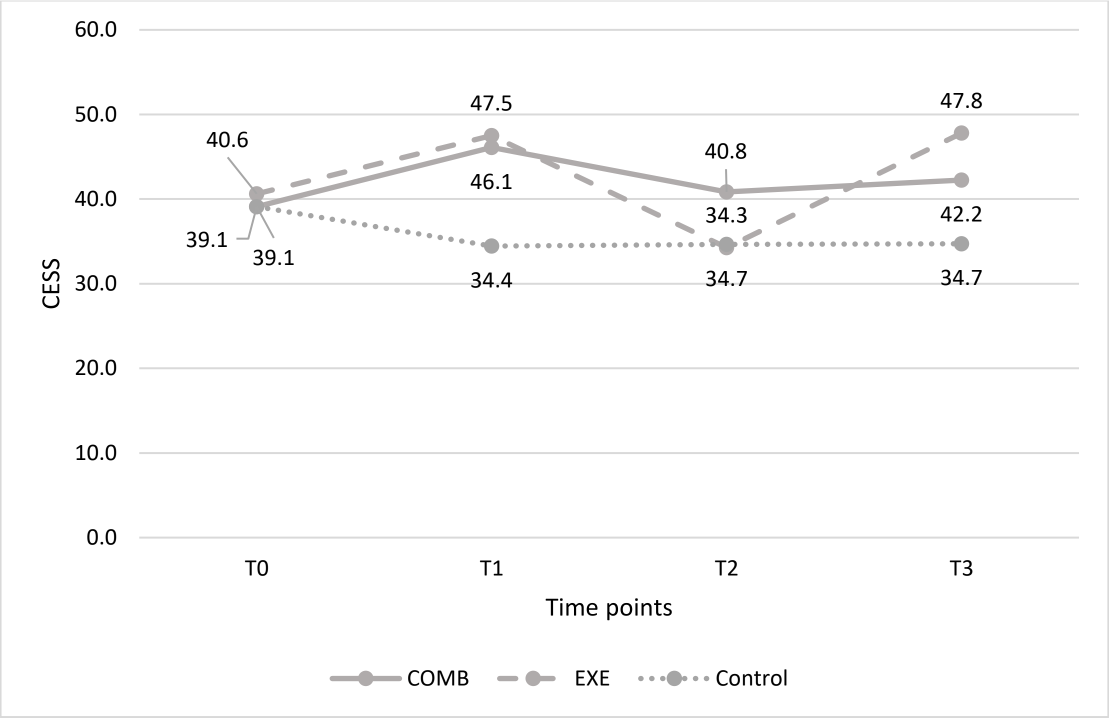

CESS: Chinese Self-Efficacy for Exercise Scale

Figure 3.6 Changes in the CESS in the three study groups over time points.

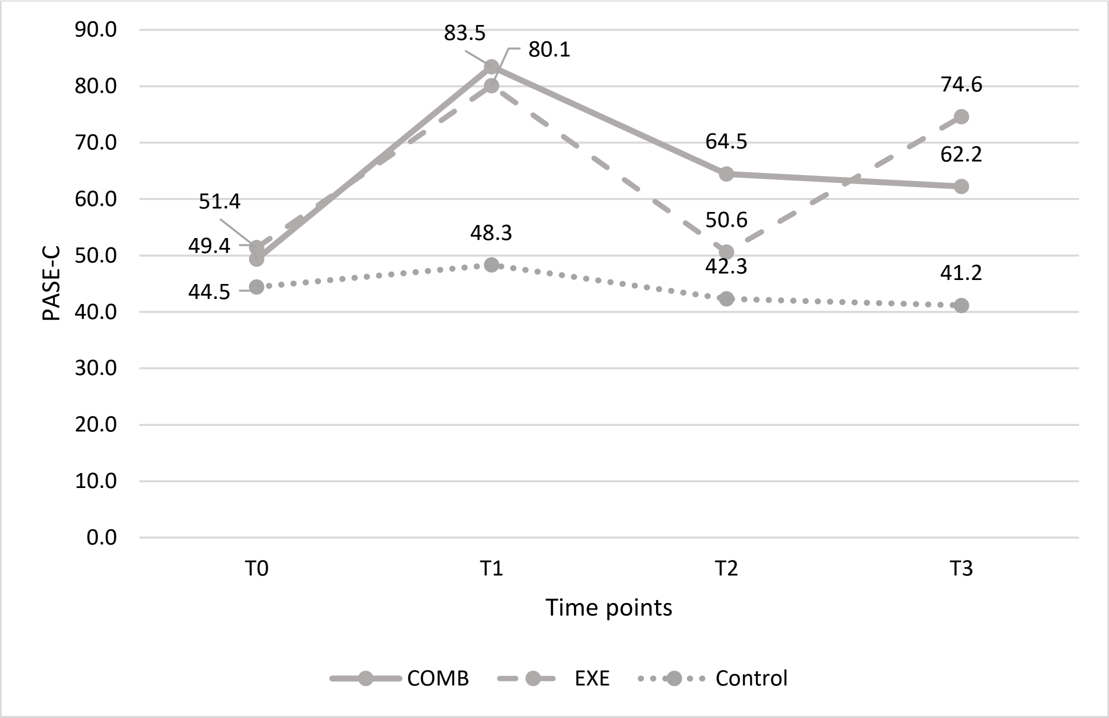

PASE-C: The Chinese Version of the Physical Activity Scale for the Elderly

Figure 3.7 Changes in the PASE-C in the three study groups over time points.

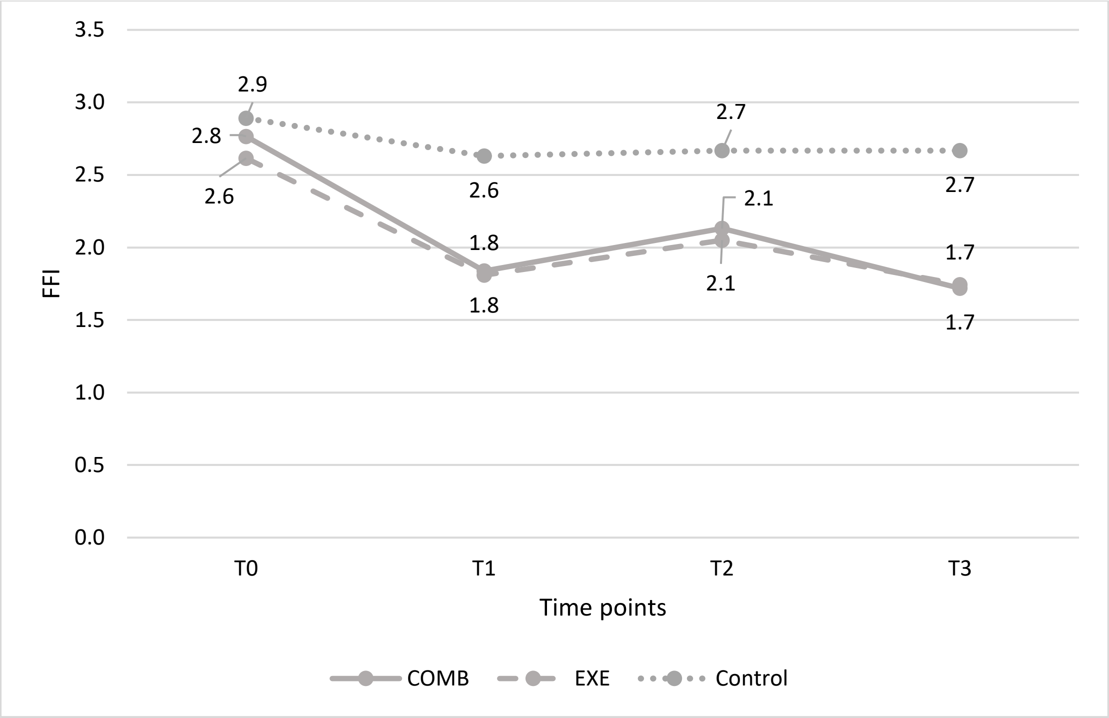

FFI: Fried Frailty Index

Figure 3.8 Changes in the FFI in the three study groups over time points.

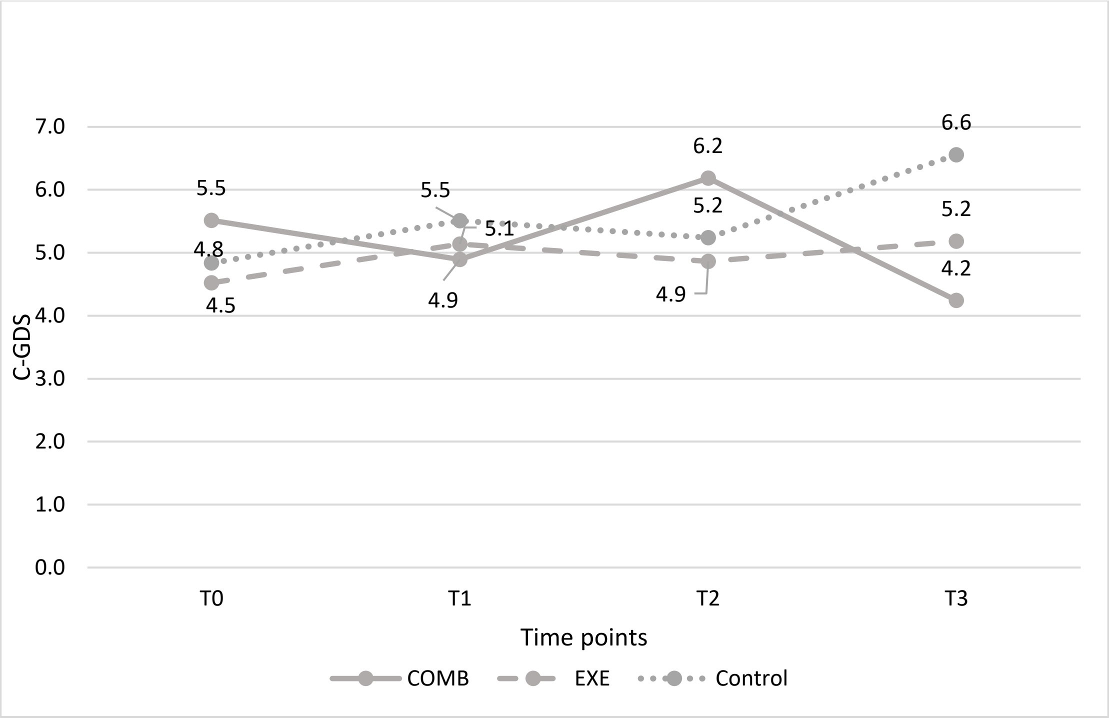

C-GDS: The Chinese Version of the Geriatric Depression Scale

Figure 3.9 Changes in the C-GDS in the three study groups over time points.

Table 1 Characteristics of the study participants

	Complete (n=122)	Incomplete (n=62)	Statistics	*P*
**Age (years)-Mean (SD)**	78.5 (6.2)	80.1 (6.6)	F= 0.750	0.94
**Gender N (%)**			X^2= 0.135	0.714
Male	12 (11)	7 (9.3)		
Female	97 (89)	68 (90.7)		
**Walking aids N (%)**			X^2= 2.277	0.893
None	50 (53.8)	34 (54)		
Walking stick	26 (28)	16 (25.4)		
Crutches	11 (11.8)	9 (14.3)		
Walking frames	1 (1.1)	1 (1.6)		
Rollators	1 (1.1)	0 (0)		
Shopping trolley	1 (1.1)	2 (3.2)		
Others	3 (3.2)	1 (1.6)		

Table 2 Outcome variables at baseline

	Complete (n=122) Mean (SD)	Incomplete (n=62) Mean (SD)	Statistics	p-value
**Primary outcome**				
Multi-dimensional fatigue inventory (CMFI-20)	66.2 (12.4)	64.9 (13.7)	F=1.178	p=0.523
**Secondary outcomes**				
Physical endurance				
Hand Grip Strength	11.3 (5.5)	11.5 (5.8)	F=0.046	p=0.843
30-s Chair Stand Test	8.4 (3.6)	8.1(3.8)	F=0.924	p=0.708
Timed-Up-and-Go test (TUG)	15.4 (8.7)	15.3 (6.3)	F=0.994	p=0.913
2-minute Walk Test	76.1 (27.3)	71.7 (26)	F=0.974	p=0.284
Exercise and activity level related				
Exercise Self-efficacy Scale (CSEE)	38.9 (16.9)	40.9 (19.4)	F=0.574	p=0.465
PASE-C	48.6 (22.4)	51 (29.5)	F=1.554	p=0.536
Frailty related				
Fried frailty index (FFI)	2.7 (0.8)	2.8 (0.7)	F=0.992	p=0.254
Psychological wellbeing related				
Geriatric depression scale (C-GDS)	4.9 (3.7)	4.8 (3.7)	F=0.017	p=0.887
